# Supplementary material for: Inter-Protein Sequence Co-Evolution Predicts Known Physical Interactions in Bacterial Ribosomes and the Trp Operon
Source: PLoS One. 2016 Feb 16;11(2):e0149166. doi: 10.1371/journal.pone.0149166 (PMC4755613; doi:10.1371/journal.pone.0149166)
Supplement: S1 Text — Multiple Sequence Alignments, Matching procedure, Inference Technique, Ribosomal Protein Interaction Partner Prediction, Artificial Data. (PDF) [file pone.0149166.s001.pdf]

INTER-PROTEIN SEQUENCE CO-EVOLUTION PREDICTS KNOWN PHYSICAL INTERACTIONS IN BACTERIAL RIBOSOMES AND THE TRP OPERON

[1]Christoph Feinauer, Department of Applied Science and Technology, and Center for Computational Sciences, Politecnico di Torino, Corso Duca degli Abruzzi 24, I-10129 Torino, Italy.
[2]Hendrik Szurmant,Department of Molecular and Experimental Medicine, The Scripps Research Institute, La Jolla, CA 92037.
[3,*]Martin Weigt, Sorbonne Universités, UPMC Univ Paris 06, UMR 7238, Computational and Quantitative Biology, 15 rue de l'Ecole de Médecine, 75006 Paris, France. CNRS, UMR 7238, Computational and Quantitative Biology, 15 rue de l'Ecole de Médecine, 75006 Paris, France.
[4, *]Andrea Pagnani, Department of Applied Science and Technology, and Center for Computational Sciences, Politecnico di Torino, Corso Duca degli Abruzzi 24, I-10129 Torino, Italy.Human Genetics Foundation-Torino, Molecular Biotechnology Center, Via Nizza 52, I-10126 Torino, Italy.

* corresponding authors

Contents

A. Multiple Sequence Alignments **2**
A.1. Multiple Sequence Alignments 2
A.2. Alignment Generation 2
A.3. Internal Sensitivity Plots 4

B. Matching Procedure **6**
B.1. Pipeline for Matching 6

C. Inference technique **7**

D. Ribosomal Protein Interaction Partner Prediction **8**
D.1. Structural view of the Ribosomal Complex 10

E. Artificial Data **18**
E.1. Monte Carlo Sequence Generation 20

A. Multiple Sequence Alignments

A.1. Multiple Sequence Alignments

The data we use are multiple sequence alignments (MSA). Each such MSA is a rectangular matrix, with entries coming from a 21-letter alphabet containing the 20 standard amino acids and a gap symbol "-". In the following we denote this alignment by a matrix

$$X = (x_i^a), \quad i = 1, ..., L, \quad a = 1, ..., M \tag{1}$$

with L being the number of residues of each MSA row, i.e., the number of residues in each considered protein, and M the number of MSA rows, i.e., the number of proteins collected in the alignment. For simplicity of notation we assume that the 21 amino acids are translated into consecutive numbers 1,...,21.

A.2. Alignment Generation

For all proteins of the small ribosomal subunit (SRU) and the large ribosomal subunit (LRU) the sequence names were extracted from the corresponding PFAM alignments [8]. Using these names, the following procedure was used to create the alignments for the single proteins:

1. Extract sequences corresponding to names from Uniprot [5]
2. Run MAFFT [11] on them using `mafft --anysymbol --auto`
3. Remove columns from the alignment that contain more than 80% gaps
4. Create an Hidden Markov Model (HMM) using `hmmbuild` from the hmmer suite [9]
5. Search Uniprot using `hmmsearch` [9]
6. Remove inserts
7. If there exist in one species two or more sequences that are more than 95% identical, remove all but one.

The number of sequences for the single files can be found in Table A

The alignments for the proteins of the Trp Operon where constructed analogously with some modifications to ensure that only full-length sequences where extracted. Also, we chose the `linsi` program of the MAFFT package to create the initial MSAs. The number of sequences for the Trp alignments can be found in Table B.

	L	M	P	S
RS2	219	6053	1.743	5.978
RS3	216	6235	1.716	7.761
RS4	171	8522	2.175	11.305
RS5	164	5075	1.678	5.845
RS6	105	4132	1.563	6.630
RS7	147	5733	1.595	4.962
RS8	127	5761	1.700	5.992
RS9	127	4983	1.663	5.917
RS10	100	4560	1.511	4.232
RS11	120	5136	1.520	4.019
RS12	124	5607	1.581	4.036
RS13	116	5729	1.856	5.763
RS14	96	5555	1.689	4.780
RS15	89	5361	1.646	6.036
RS16	83	4463	1.507	5.851
RS17	82	4774	1.616	5.481
RS18	73	4512	1.483	4.879
RS19	89	5364	1.537	4.700
RS20	88	3848	1.676	7.460
RS21	65	3209	1.456	4.188

	L	M	P	S
RL3	205	6077	2.025	6.522
RL4	198	5671	1.906	6.810
RL5	177	5032	1.636	6.245
RL6	178	5308	1.765	6.894
RL9	149	4199	1.698	7.621
RL11	141	5027	1.683	5.517
RL13	147	5091	1.717	6.458
RL14	120	5145	1.528	4.358
RL15	140	5926	1.964	6.754
RL16	133	5673	1.604	4.904
RL17	121	4345	1.612	7.637
RL18	111	4961	1.674	6.570
RL19	116	4079	1.511	6.454
RL20	119	4476	1.554	5.864
RL21	102	4123	1.551	6.486
RL22	108	6378	1.918	5.790
RL23	87	5632	1.711	6.292
RL24	99	9062	3.073	12.820
RL25	186	3272	1.680	6.109
RL27	89	3989	1.486	5.419
RL28	74	4051	1.584	5.694
RL29	66	4456	1.540	6.024
RL30	60	4356	1.671	5.313
RL32	60	4206	1.463	4.997
RL33	49	4604	1.678	4.943
RL34	45	3195	1.346	4.280
RL35	65	3691	1.502	5.889
RL36	38	3779	1.408	3.103

Table A: Alignment sizes (M) and lengths (L) for proteins of the small (RSXX) and large (RLXX) ribosomal subunit. (P) indicates the average number of paralogs per species and (S) the standard deviation of this number.

	L	M	P	S
TrpA	259	10220	4.457	32.604
TrpB	399	46557	16.992	145.826
TrpC	254	10323	4.536	39.868
TrpD	337	17582	7.130	59.693
TrpE	460	28173	11.749	124.933
TrpF	197	8713	4.122	32.400
TrpG	192	78265	24.713	187.331

Table B: Alignment sizes (M) and lengths (L) for proteins of the Trp Operon. (P) indicates the average number of paralogs per species and (S) the standard deviation of this number.

A.3. Internal Sensitivity Plots

As an assessment of quality for the alignments, sensitivity plots using the pdb files `2Z4K` and `2Z4L` were made. Figure A shows results for contact predictions based on the GaussDCA [2] and plmDCA alghorithm [7].

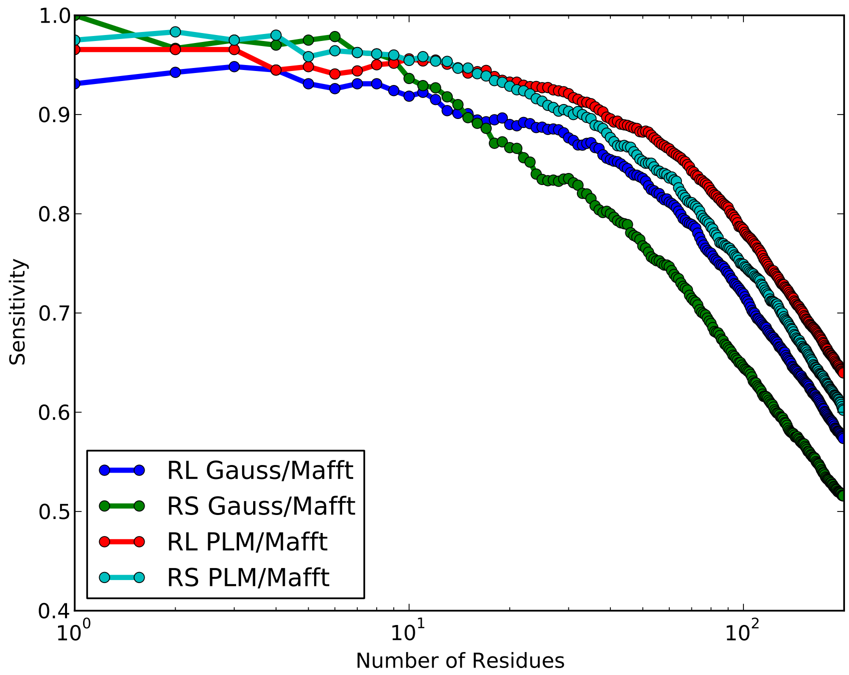

Figure A: Intra-Protein Sensitivity Plots. On the alignments for the single ribosomal proteins the plmDCA algorithm was run and an ordered list of residue pairs obtained. For every number n on the abscissae the fraction of the number of true positives (the sensitivity) in the first n pairs on this list was calculated for every protein. The plot shows the mean of these values for the Gaussian algorithm of [2] and the plmDCA algorithm run on the proteins of the large and small ribosomal subunit.

B. Matching Procedure

B.1. Pipeline for Matching

The problem of generating a concatenated alignment from two MSAs of two different protein families (say MSA_1 and MSA_2) is to decide which sequence from the first alignment should be concatenated to which sequence from the other alignment. This means to find for any protein p_i^1 in MSA_1 a matching partner p_j^2 in MSA_2 belonging to the same species. The problem is trivially solved in the case when no paralogs are present and each species has one and only one sequence in each individual MSA. In this case we can simply concatenate these two sequences (we term this case *matching by uniqueness*). The problem is that species often have several paralogs. In this case, given that we would like to observe a co-evolutionary signal between protein interaction partners, one would like to match sequences of proteins that are (possibly) interacting.

As long as Prokaryotes are concerned, it turns out empirically that proteins are more likely to interact if their genes are *co-localized* on the DNA [15, 4]. This suggests to try to match proteins that are close on the genome when creating a concatenated MSA.

As a proxy to the genomic distance we use a *distance* between Uniprot accession numbers (UAN). This UAN consists of a 6 digit alphanumeric sequence for every sequence and can be extracted from the sequence annotation, e.g. the "`D8UHT6`" part of the sequence annotation "`D8UHT6_PANSA`".

We define the distance between UANs as follows: Different positions in the UAN can take on different values, some only numeric (0-9) and some alphanumeric values (0-9,A-Z). We define for every position $i \in 1 \dots 6$ the number B_i as the number of different values position i can take, i.e. $B_i = 10$ for the numeric positions and $B_i = 36$ for the alphanumeric positions.

We further map the possible single position values in the UAN to the natural numbers in ascending order, i.e. we assign to the numeric symbols $0-9$ the natural numbers $0-9$ and to the letters the natural numbers following 9 (so to A we assign 10, to B we assign 11 etc.). This leads for example for the the UAN `L9XG27` to the numeric sequence $A = (21, 9, 33, 16, 2, 7)$.

Now we can define a unique number N for any UAN that has been mapped to the sequence of natural numbers A_i as

$$N = A_6 + \sum_{i=1}^{5} A_i \left(\prod_{j=i+1}^{6} B_j \right) \tag{2}$$

The distance between two UANs that have been mapped to the numbers N_1 and N_2 can now be defined as

$$D_{12} = |N_1 - N_2| \tag{3}$$

This procedure induces a distance D_{ij} for any sequence $p_i \in \text{MSA}_1$ and $p_j \in \text{MSA}_2$, where both p_i, p_j belong to the same species. In this way we define a complete weighted bipartite graph, and the problem of finding the proper pairing can thus be translated

into a minimum weighted bipartite matching problem. This problem can be readily solved using a standard linear programming techniques. Finally we discard from the optimal solution sequence pairs whose distance is above a given threshold of 100 (manually optimized on the small ribosomal subunit). In the cases we analyzed, such a threshold moderately increases the quality of the prediction of interaction partners.

C. Inference technique

As a simple but meaningful statistical model, we consider a pairwise generalized 21 states (to mimic the 20 amino acids + 1 insert symbol alphabet of MSAs) Potts model with the following Hamiltonian

$$\mathcal{H} = - \sum_{0 \leq i < j \leq L} J_{i,j}(x_i, x_j) - \sum_{i=1}^{L} h_i(x_i) \tag{4}$$

We can now assume to have a dataset $D = \{x^1, \ldots, x^M\}$, where x represents one sequence, either artificially generated, or extracted using the bioinformatic pipeline discussed above. Notice that if the sequences x are concatenations of two sequences (x, x'), the sums in Equation 4 can be split into three parts: One in which appear only sites in x, one in which appear only sites in x' and one interaction part with J_{ij} for which i is in x and j in x'. By labeling the first part $H(x)$, the second $H'(x')$ and the third $H^{int}(x, x')$ one arrives at the representation referred to in the main text. Given that the representations are mathematically equivalent, we will here in supplemental information treat the sequence as one simple sequence x.

The inference proceeds by assuming as a working hypothesis that the dataset D is composed by configuration sampled uniformly from the equilibrium Boltzmann-Gibbs distribution $P(\vec{x}) = \exp(-\mathcal{H})/Z$ (as an inference process, we are free to consider $T = \beta = 1$). We are now ready to use D to infer the topology of the network. To do so – as discussed in the main text – in the last years different maximum-likelihood techniques have been proposed [16, 14, 12, 1, 10, 6]. So far the most promising in terms of accuracy seems to be the pseudo-likelihood maximization introduced in [6] where from the previously defined Boltzmann-Gibbs measure we consider the following conditional probability distribution:

$$P_i(x_i | x_{\setminus i}) = \frac{\exp\left(\sum_{j \neq i} J_{ij}(x_i, x_j) + h_i(x_i)\right)}{\sum_{a=1}^{21} \exp\left(\sum_{j \neq i} J_{ij}(x_i, a) + h_i(a)\right)} \tag{5}$$

Given a data set D we can thus maximize the conditional likelihood by maximizing

$$L_i(J_{i,\setminus i}, h_i) = \frac{1}{M} \sum_{\alpha=1}^{M} \log P_i(x_i^\alpha | x_{\setminus i}^\alpha) \quad , \tag{6}$$

as a function of $J_{i,\setminus i}, h_i$. As customary in many maximum-likelihood inference techniques, we add to the maximization an $\mathcal{L}_2$ regularization term, so that eventually the

extremization procedure turns out to be:

$$\{J^*_{i,\setminus j}, h^*_i\} = \underset{J_{i,\setminus i}, h_i}{\operatorname{argmax}} \{L_i - \lambda_J \sum_{j \neq i} \|J_{ij}\|_2 - \lambda_h \|h_i\|_2\}\,, \tag{7}$$

with $\|J_{ij}\|_2 = \sum_{a,b=1}^{21} J_{ij}^2(a,b)$, and $\|h_i\|_2 = \sum_{a=1}^{21} h^2(a)$. We refer to the original paper [6] for the details of the implementation. We only mention that beside the original MATLAB [13] implementation available at `http://plmdca.csc.kth.se/`, we developed an efficient implementation of the pseudo-likelihood implementation in a new open-source language called Julia [3]. The package can be downloaded at `https://github.com/pagnani/PlmDCA`.

D. Ribosomal Protein Interaction Partner Prediction

Using the ribosomal alignments as described in Section A and the matching as described in Section B, concatenated alignments for the ribosomal proteins (small and large ribosomal subunit independently) were created. Table C shows the resulting alignment sizes for the SRU and Table E for the LRU.

The creation of the alignments for the Trp Proteins was analagous and the resulting alignment sizes can be found in Table G.

As discussed in the main text, in principle one would be interested in a MSA in which a sequence is a concatenation of sequences from all proteins families in the complex at once. A comparative glance at Tables E and A shows that in the matching procedure described above a lot of sequences have to be discarded for not having a suitable matching partner. This leads to a reduction of the predictive power of the method. It is expected that extending the matching procedure to more than two proteins would lead to very low sequence numbers in the matched alignments and in turn reduce the predictive power of the method further. For this reason we only performed the concatenation of pairs of proteins.

	RL2	RL3	RL4	RL5	RL6	RL7	RL8	RL9	RL10	RL11	RL12	RL13	RL14	RL15	RL16	RL17	RL18	RL19	RL20	RL21
RL2		2914	2537	2458	2224	2825	2833	2491	2457	2839	2664	2342	2511	2748	2462	2373	2515	2842	2109	1740
RL3			2947	2719	2430	3109	3223	2531	2680	3097	2922	2577	2992	2694	2645	2686	2659	3213	2123	1907
RL4				2411	1837	2719	2812	2214	2314	2802	2528	2463	2522	2319	2064	2354	2182	2765	1774	1468
RL5					2231	2613	2736	2508	2607	2623	2410	2532	2517	2381	2221	2699	2142	2657	2127	1743
RL6						2206	2251	2216	2200	2204	2041	2117	1938	2169	2430	2226	2590	2263	2116	1931
RL7							3001	2469	2580	2914	3172	2452	2753	2650	2414	2524	2483	2937	2089	1711
RL8								2539	2782	3098	2831	2654	3004	2707	2494	3037	2497	3402	2114	1786
RL9									2466	2564	2348	2400	2284	2383	2204	2469	2188	2489	2103	1755
RL10										2579	2423	2460	2443	2378	2212	2711	2144	2784	2100	1734
RL11											2810	2618	2849	2694	2417	2604	2497	3008	2083	1729
RL12												2295	2646	2507	2224	2369	2303	2828	1925	1542
RL13													2395	2188	2174	2502	2117	2564	2060	1712
RL14														2420	2169	2510	2398	2920	1804	1529
RL15															2417	2348	2461	2679	2115	1753
RL16																2212	2532	2474	2116	1925
RL17																	2127	2918	2097	1735
RL18																		2484	2043	1867
RL19																			2096	1767
RL20																				1683
RL21																				
	2520	2740	2370	2439	2191	2612	2726	2348	2424	2633	2463	2349	2453	2422	2306	2447	2328	2689	2036	1738

Table C: Matched Alignment Sizes for Small Ribosomal Subunit, at threshold 100

	RL2	RL3	RL4	RL5	RL6	RL7	RL8	RL9	RL10	RL11	RL12	RL13	RL14	RL15	RL16	RL17	RL18	RL19	RL20	RL21
RL2		2594	2143	2343	2149	2608	2611	2342	2333	2592	2379	2095	2256	2533	2318	2303	2311	2599	2051	1692
RL3			2219	2373	2371	2615	2628	2363	2348	2579	2406	2097	2267	2535	2506	2341	2444	2656	2057	1871
RL4				1895	1722	2178	2140	1893	1888	2117	2010	1707	1886	2072	1877	1858	1877	2146	1653	1394
RL5					2156	2356	2364	2344	2333	2322	2156	2078	1984	2313	2160	2320	2084	2319	2069	1707
RL6						2135	2189	2153	2146	2134	1960	2063	1840	2138	2376	2150	2251	2180	2071	1879
RL7							2617	2327	2326	2596	2494	2088	2267	2536	2304	2304	2310	2605	2043	1665
RL8								2338	2341	2623	2379	2113	2302	2570	2385	2336	2333	2669	2057	1743
RL9									2323	2324	2156	2071	1996	2315	2155	2303	2102	2320	2057	1700
RL10										2327	2153	2090	1996	2301	2159	2302	2096	2330	2055	1693
RL11											2386	2091	2280	2559	2318	2291	2318	2596	2040	1685
RL12												1920	2145	2324	2094	2120	2069	2395	1866	1508
RL13													1806	2077	2091	2052	2054	2086	2003	1661
RL14														2213	2037	1980	2109	2290	1735	1485
RL15															2316	2287	2304	2539	2043	1697
RL16																2149	2451	2373	2066	1877
RL17																	2077	2321	2047	1687
RL18																		2308	1998	1827
RL19																			2033	1734
RL20																				1617
RL21																				
	2329	2383	1930	2193	2109	2335	2355	2189	2186	2325	2154	2013	2046	2299	2211	2170	2175	2342	1977	1691

Table D: Matched Alignment Sizes for Small Ribosomal Subunit, at threshold 0 (matching by uniqueness)

	RL2	RL3	RL4	RL5	RL6	RL9	RL11	RL13	RL14	RL15	RL16	RL17	RL18	RL19	RL20	RL21	RL22	RL23	RL24	RL25	RL27	RL28	RL29	RL30	RL32	RL33	RL34	RL35	RL36
RL2		2699	2720	2875	2824	2142	2505	2461	3077	2658	3101	2438	2672	2190	2509	2174	2957	3075	2435	1739	2164	1932	2904	2471	2296	2163	1970	2094	2328
RL3			2789	2626	2923	2149	2382	2395	2873	2604	2626	2456	2649	2184	2161	2164	3132	2661	2338	1733	2184	1964	2591	2290	2033	1902	1993	2108	1984
RL4				2639	2709	2167	2407	2418	2637	2676	2647	2438	2871	2209	2167	2168	2788	2695	2805	1747	2195	1962	2652	2333	2040	1894	2001	2134	2011
RL5					2902	2232	2492	2498	2799	2692	3134	2608	2775	2312	2327	2309	2688	3035	2483	1773	2299	2014	2744	2389	2164	1945	2084	2203	2136
RL6						2216	2551	2506	3043	2768	2839	2651	2828	2283	2277	2275	2990	2773	2495	1785	2286	2005	2828	2455	2114	1937	2039	2207	2101
RL9							2154	2156	2168	2161	2174	2191	2238	2283	2224	2237	2153	2165	502	1792	2259	2025	2230	1877	2099	1810	2106	2190	1768
RL11								2422	2492	2375	2468	2223	2499	2217	2179	2174	2370	2539	2314	1732	2187	1973	2457	2131	2040	2024	1991	2133	1777
RL13									2491	2482	2498	2246	2493	2208	2197	2198	2340	2482	1127	1755	2217	1980	2445	2110	2053	1852	1999	2155	1800
RL14										2643	3080	2465	2752	2232	2574	2227	3166	3012	2328	1735	2208	1989	2606	2241	2345	2181	2003	2126	2345
RL15											2616	2509	2740	2189	2169	2160	2714	2700	2354	1760	2196	1964	2706	2388	2024	1848	1970	2109	2040
RL16												2488	2730	2240	2564	2229	2812	3348	2314	1759	2213	1991	2610	2259	2372	2191	2012	2142	2325
RL17													2755	2385	2176	2341	2465	2530	2207	1726	2380	2146	2689	2180	2190	1917	2131	2181	2302
RL18														2422	2223	2369	2734	2739	2934	1772	2417	2170	2886	2454	2227	1975	2176	2216	2193
RL19															2331	2437	2188	2262	580	1774	2507	2277	2434	1913	2361	1906	2225	2315	1948
RL20																2311	2483	2518	411	1787	2297	2011	2248	1868	2450	2161	2048	2477	2152
RL21																	2202	2242	542	1754	2692	2163	2380	1887	2258	1890	2177	2259	1913
RL22																		2942	2380	1739	2208	1970	2595	2251	2297	2160	1989	2120	2294
RL23																			2405	1748	2254	2007	2727	2397	2381	2221	2044	2152	2337
RL24																				391	503	528	2459	2093	449	1111	522	437	1468
RL25																					1770	1595	1745	1547	1649	1564	1598	1761	1362
RL27																						2234	2427	1915	2300	1928	2232	2295	1931
RL28																							2148	1719	2185	1935	2015	2039	1750
RL29																								2584	2223	1957	2163	2251	2160
RL30																									1765	1579	1732	1851	1738
RL32																										2183	2074	2130	2132
RL33																											1741	1819	1921
RL34																												2089	1779
RL35																													1800
RL36																													
	2485	2378	2390	2471	2486	2067	2257	2214	2494	2365	2492	2336	2497	2172	2189	2148	2469	2514	1604	1664	2168	1953	2459	2086	2101	1918	1961	2064	1993

Table E: Matched Alignment Sizes for Large Ribosomal Subunit, at threshold 100

	RL2	RL3	RL4	RL5	RL6	RL9	RL11	RL13	RL14	RL15	RL16	RL17	RL18	RL19	RL20	RL21	RL22	RL23	RL24	RL25	RL27	RL28	RL29	RL30	RL32	RL33	RL34	RL35	RL36
RL2		2144	2173	2333	2307	2079	2277	2286	2568	2115	2547	2041	2313	2120	2325	2095	2325	2552	217	1698	2100	1895	2257	1964	2182	1875	1918	2052	1900
RL3			2139	2179	2162	2087	2152	2169	2165	2102	2168	2033	2178	2126	2109	2095	2112	2177	177	1703	2115	1899	2140	1851	1965	1651	1933	2068	1648
RL4				2205	2188	2099	2175	2191	2190	2118	2197	2045	2207	2140	2119	2102	2117	2193	189	1704	2126	1912	2170	1865	1978	1663	1944	2075	1652
RL5					2425	2176	2316	2319	2388	2151	2370	2162	2379	2255	2257	2221	2150	2369	221	1735	2235	1960	2394	2038	2093	1727	2005	2161	1771
RL6						2164	2307	2310	2344	2149	2337	2134	2368	2221	2214	2187	2130	2324	221	1725	2204	1949	2379	2016	2045	1694	1981	2144	1713
RL9							2088	2106	2110	2095	2122	2142	2178	2232	2176	2187	2102	2119	167	1735	2224	1967	2181	1824	2059	1697	2018	2147	1720
RL11								2305	2317	2117	2300	2061	2319	2143	2129	2115	2109	2299	219	1693	2133	1922	2289	1978	1994	1672	1938	2074	1668
RL13									2312	2130	2306	2064	2323	2152	2141	2129	2121	2305	205	1713	2145	1918	2292	1984	1988	1670	1952	2090	1668
RL14										2146	2600	2089	2349	2165	2392	2155	2388	2606	217	1710	2151	1940	2318	2012	2259	1941	1961	2085	1998
RL15											2166	2062	2162	2137	2120	2107	2120	2132	181	1713	2127	1917	2110	1842	1975	1657	1936	2073	1653
RL16												2089	2335	2171	2370	2144	2347	2539	216	1724	2155	1935	2304	2001	2226	1902	1963	2095	1931
RL17													2302	2346	2146	2280	2049	2121	222	1677	2337	2099	2305	1798	2155	1724	2094	2144	1802
RL18														2366	2177	2310	2144	2381	293	1731	2366	2127	2521	2056	2177	1774	2126	2166	1821
RL19															2260	2370	2138	2208	235	1748	2438	2156	2392	1875	2248	1816	2190	2260	1889
RL20																2231	2345	2379	174	1737	2226	1960	2197	1840	2294	1952	2003	2177	2011
RL21																	2125	2179	224	1720	2367	2089	2317	1838	2191	1777	2130	2203	1849
RL22																		2373	170	1707	2132	1917	2107	1810	2215	1908	1946	2081	1936
RL23																			227	1716	2187	1955	2351	2006	2289	1957	1988	2107	1999
RL24																				116	238	211	288	169	224	180	207	182	195
RL25																					1733	1539	1713	1517	1602	1474	1566	1704	1323
RL27																						2164	2376	1863	2243	1814	2194	2236	1879
RL28																							2109	1654	2036	1697	1980	1989	1685
RL29																								2052	2188	1771	2132	2210	1835
RL30																									1724	1427	1711	1821	1441
RL32																										1988	2048	2084	2033
RL33																											1655	1708	1693
RL34																												2051	1730
RL35																													1763
RL36																													
	2095	1980	1996	2107	2084	2000	2040	2046	2138	1975	2127	2019	2141	2100	2088	2062	2040	2144	207	1613	2090	1878	2132	1785	2018	1695	1904	1998	1722

Table F: Matched Alignment Sizes for Large Ribosomal Subunit, at treshold 0 (matching by uniqueness)

In order to produce an interaction score for the two proteins, we run the PLM algorithm [6] on the concatenated alignments. This results in a list of residue pairs of the alignment ordered by their interaction strength. We filtered out the pairs that contain one residue of one protein and one of the other. This results in a list of *possibly* interacting inter-protein residue pairs ordered by the interaction score. In order to arrive at an interaction score for the two proteins we took the mean of the scores for the 4 highest scoring pairs (PPI-score). The number 4 was used because it performed best on the small ribosomal subunit, but the predictive performance on a larger-scale network is virtually identical for any value between 1 and 6 (see Figure S8). The list of protein pairs ordered by this score was used for prediction. The first few predictions are shown in Table H. For completeness, we show the same table but with the score calculated by the Gaussian approximation of [2] in Table I. Finally in Table K we display for the LSU the number of intra/inter-protein contacts, while in Table L we do the same for the LRU.

Table J shows the interaction scores for the protein pairs of the Trp Operon.

D.1. Structural view of the Ribosomal Complex

In Fig. C we display a cartoon view of the ribosomal protein network. The contact map for the the small and large ribosomal units are displayed in Fig. D

P1	P2	tr=100	tr=0
TrpC	TrpG	4272	18
TrpE	TrpF	2519	830
TrpA	TrpD	2823	743
TrpD	TrpG	6249	28
TrpB	TrpF	3643	95
TrpB	TrpD	3737	95
TrpB	TrpG	8053	41
TrpE	TrpG	5324	8
TrpD	TrpF	2819	695
TrpC	TrpF	3825	1578
TrpA	TrpC	3198	1546
TrpC	TrpD	3392	748
TrpA	TrpF	3357	1433
TrpA	TrpE	3118	905
TrpD	TrpE	2681	482
TrpB	TrpC	3326	82
TrpB	TrpE	3911	53
TrpC	TrpE	2976	930
TrpF	TrpG	3635	32
TrpA	TrpB	4374	95
TrpA	TrpG	4646	22

Table G: Matched Alignment Sizes for Trp for different matching thresholds (threshold 0 corresponds to matching by uniqueness)

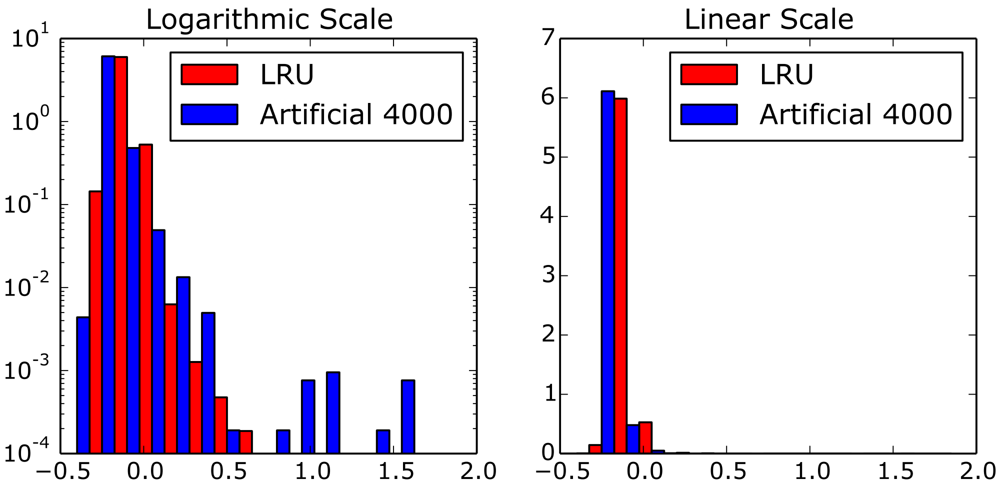

Figure B: Histograms of interaction scores resulting from the analysis of the LRU and the artificial complex (combined strategy). Both intra- and inter-protein scores are included. The plots are normalized such that the area of all bars of a given color sums to one. The data is shown both on a logarithmic (left) and on a linear scale (right).

P1	P2	Score	Interacting	P1	P2	Score	Interacting
RS10	RS14	0.618890	1	RL20	RL21	0.576795	1
RS18	RS6	0.422457	1	RL14	RL19	0.514107	1
RS14	RS3	0.394753	1	RL15	RL35	0.440323	1
RS10	RS9	0.347508	1	RL15	RL21	0.439233	1
RS13	RS19	0.317640	1	RL17	RL32	0.425920	1
RS13	RS21	0.306248	0	RL20	RL32	0.421733	1
RS11	RS21	0.296700	1	RL23	RL29	0.414060	1
RS14	RS19	0.291335	1	RL13	RL20	0.334348	1
RS12	RS21	0.290965	0	RL19	RL3	0.328640	1
RS16	RS4	0.287438	0	RL30	RL34	0.326368	0
RS21	RS7	0.287102	0	RL22	RL32	0.324540	1
RS13	RS15	0.284783	0	RL16	RL36	0.318915	1
RS12	RS16	0.283105	0	RL16	RL33	0.313083	0
RS19	RS21	0.282142	0	RL33	RL36	0.307188	0
RS10	RS18	0.279595	0	RL27	RL34	0.306283	0

Table H: Ordered List of Interaction Candidates SRU (left) and LRU (right) based on plmDCA scores; the fourth column indicates whether the protein pair is indeed interacting

P1	P2	Score	Interacting
RS10	RS9	1.123465	1
RS10	RS14	1.102428	1
RS12	RS21	1.079407	0
RS13	RS18	1.029537	0
RS14	RS17	1.001716	0
RS12	RS15	0.997813	0
RS18	RS6	0.963688	1
RS11	RS13	0.943144	0
RS19	RS21	0.942921	0
RS15	RS18	0.938286	0
RS14	RS15	0.933949	0
RS13	RS15	0.933337	0
RS13	RS19	0.918528	1
RS18	RS21	0.918101	1
RS10	RS13	0.917482	0

P1	P2	Score	Interacting
RL20	RL21	1.665182	1
RL14	RL19	1.430611	1
RL15	RL21	1.333611	1
RL15	RL35	1.134808	1
RL23	RL29	1.086992	1
RL20	RL32	1.037364	1
RL22	RL32	1.029724	1
RL30	RL34	1.008776	0
RL17	RL32	1.002790	1
RL34	RL36	0.983223	0
RL21	RL2	0.977507	0
RL21	RL34	0.958441	0
RL18	RL34	0.942494	0
RL36	RL6	0.925895	1
RL33	RL36	0.898444	0

Table I: Ordered List of Interaction Candidates SRU (left) and LRU (right) based on Gaussian scores; the fourth column indicates whether the protein pair is indeed interacting

TrpA	TrpB	0.375
TrpE	TrpG	0.295
TrpA	TrpC	0.167
TrpA	TrpF	0.162
TrpC	TrpF	0.146
TrpA	TrpD	0.144
TrpC	TrpD	0.141
TrpB	TrpF	0.136
TrpC	TrpE	0.135
TrpD	TrpF	0.135
TrpB	TrpC	0.132
TrpA	TrpE	0.126
TrpC	TrpG	0.121
TrpB	TrpD	0.120
TrpE	TrpF	0.115
TrpD	TrpE	0.107
TrpF	TrpG	0.107
TrpA	TrpG	0.104
TrpD	TrpG	0.100
TrpB	TrpE	0.096
TrpB	TrpG	0.071

Table J: Ordered List of Interaction Scores for the Trp Operon based on plmDCA scores

SRU Intra-Protein		
	SEP=0	SEP=5
RS2	2337	1610
RS3	2217	1494
RS4	1728	1152
RS5	1684	1175
RS6	1002	666
RS7	1494	982
RS8	1334	903
RS9	1240	799
RS10	878	557
RS11	1220	822
RS12	1136	731
RS13	1024	623
RS14	790	440
RS15	823	489
RS16	685	436
RS17	733	487
RS18	482	293
RS19	748	482
RS20	792	464
RS21	297	110
SUM:	22644	14715

SRU Inter-Protein		
RS2	RS5	4
RS2	RS8	3
RS3	RS5	17
RS3	RS10	105
RS3	RS14	209
RS4	RS5	84
RS5	RS8	120
RS6	RS18	150
RS7	RS9	19
RS7	RS11	46
RS8	RS12	12
RS8	RS17	28
RS9	RS10	28
RS9	RS14	7
RS10	RS14	150
RS11	RS18	20
RS11	RS21	199
RS12	RS17	34
RS13	RS19	80
RS14	RS19	50
RS18	RS21	36
SUM:		1401
FRACTION	SEP=0	0.058
FRACTION	SEP=5	0.087

Table K: Left table: number of intra-protein contacts below 8Å of all residues (SEP=0 column), and considering only those with a distance on the sequence of at least 5 residues (SEP = 5 column) for the SRU. Right table: number of inter-protein contacts below 8Åfor the SRU. Fractions are defined as $\frac{\#Intra}{\#Intra+\#Inter}$ where $\#Inter$ is computed assuming SEP=0,5 respectively.

LRU Intra-Protein		
	SEP=0	SEP=5
RL32	324	157
RL33	399	256
RL34	303	145
RL35	495	268
RL36	332	208
RL2	2687	1801
RL3	1931	1263
RL4	1869	1199
RL5	1887	1257
RL6	1811	1217
RL9	1360	855
RL11	1390	903
RL13	1464	959
RL14	1266	869
RL15	920	481
RL16	1343	915
RL17	1194	767
RL18	1150	777
RL19	1043	669
RL20	1045	600
RL21	915	600
RL22	1085	720
RL23	735	461
RL24	386	233
RL25	893	597
RL27	692	442
RL29	538	303
RL30	511	321
RL28	587	351
SUM:	30555	19594

LRU Inter-Protein		
RL32	RL17	78
RL32	RL20	17
RL32	RL22	73
RL33	RL35	21
RL35	RL15	149
RL35	RL27	1
RL36	RL6	10
RL36	RL16	1
RL3	RL13	20
RL3	RL14	34
RL3	RL17	21
RL3	RL19	123
RL4	RL15	83
RL4	RL20	6
RL9	RL28	63
RL13	RL20	118
RL13	RL21	8
RL14	RL19	191
RL15	RL20	2
RL15	RL21	24
RL16	RL25	53
RL16	RL27	9
RL17	RL22	12
RL18	RL27	12
RL20	RL21	229
RL23	RL29	81
SUM:		1439
FRACTION	SEP=0	0.045
FRACTION	SEP=5	0.068

Table L: Left table: number of intra-protein contacts below 8Å of all residues (SEP=0 column), and considering only those with a distance on the sequence of at least 5 residues (SEP = 5 column) for the LRU. Right table: number of inter-protein contacts below 8Åfor the LRU. Fractions are defined as $\frac{\#Intra}{\#Intra+\#Inter}$ where $\#Inter$ is computed assuming SEP=0,5 respectively.

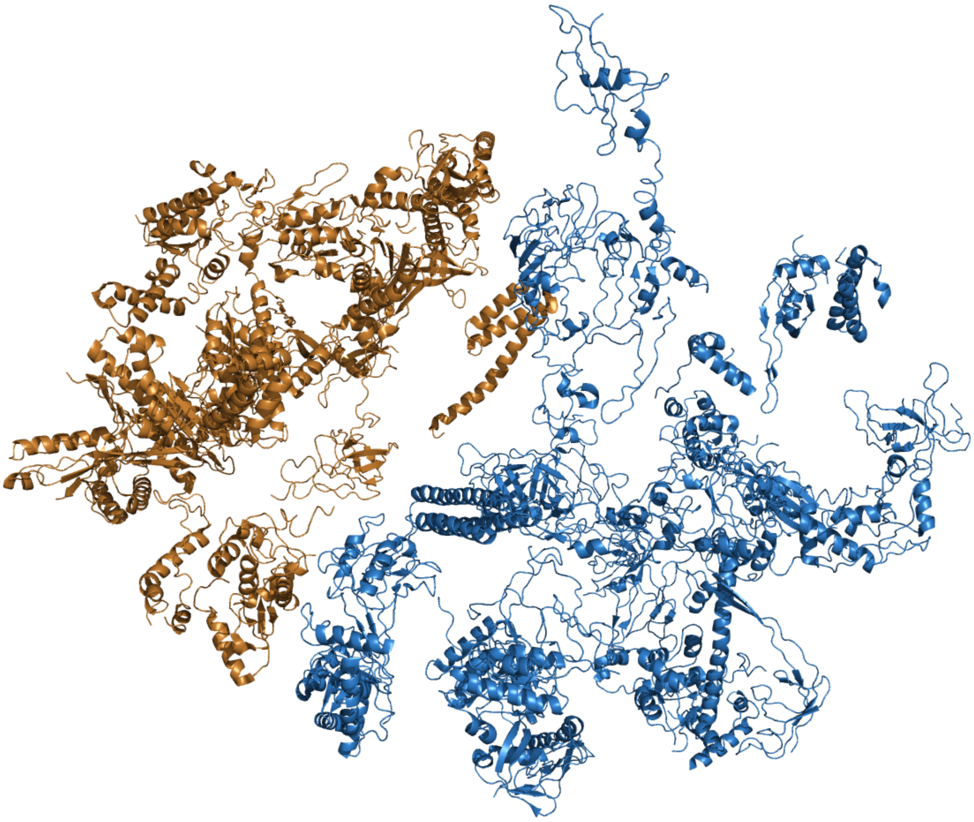

Figure C: Cartoon view of the small (brass color) and large (blue color) bacterial ribosomal complexes 2Z4K, 2Z4L. For the ease of visualization we have carved out the ribosomal RNAs strands.

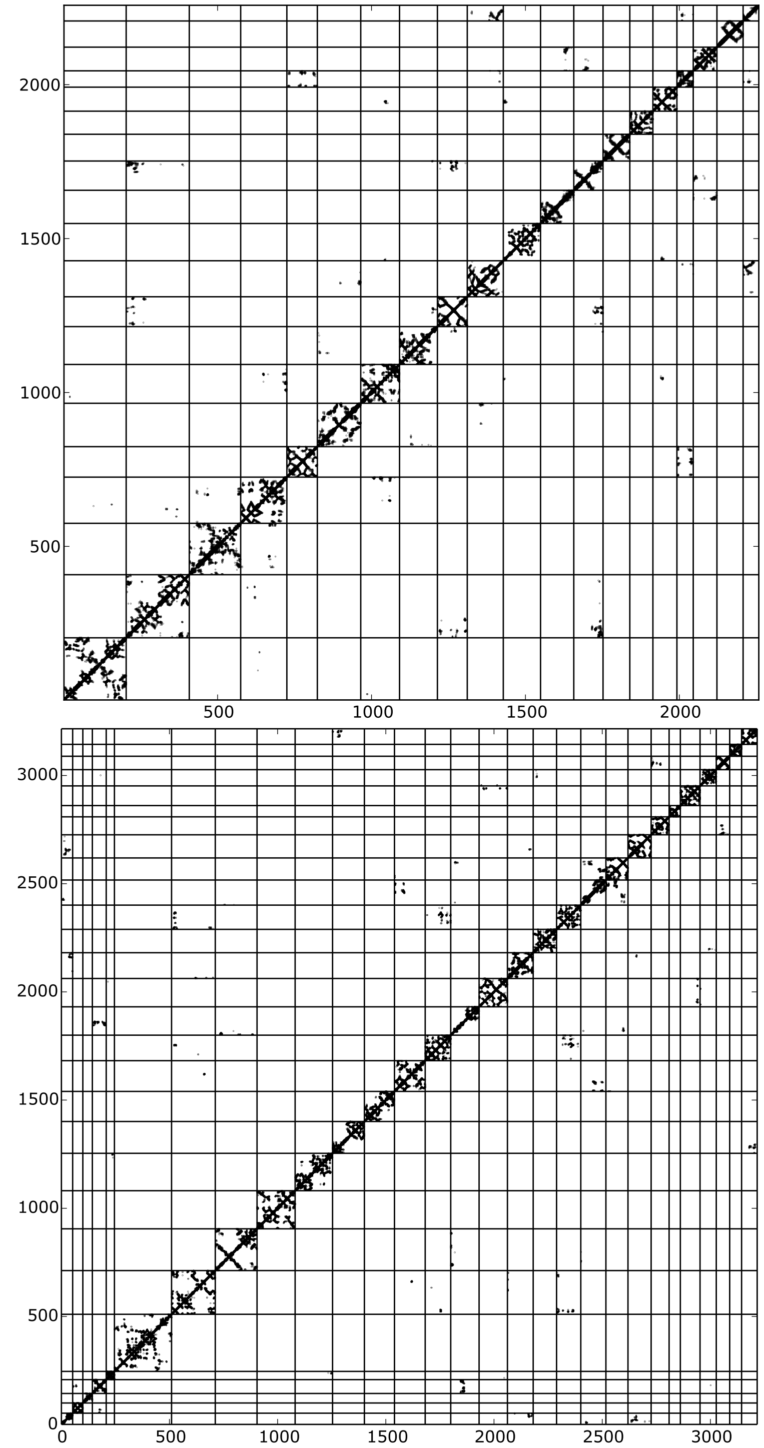

Figure D: Upper panel: contact map of the SRU (threshold distance 8Å). Lower panel: contact map of the LRU.

E. Artificial Data

An artificial large network consisting of 5 proteins was created in two steps:

1) First, a contact map was defined. This contact map contains the information which residues are in contact. This includes internal residue contacts (where both residues belong to one of the 5 proteins) and inter-protein residue contacts (where one residue belongs to one protein and the other to a different protein). The contact map is therefore a binary, symmetric matrix of size $N_{all} \times N_{all}$ with $N_{all} = N_1 + N_2 + N_3 + N_4 + N_5$ where N_i is the number of residues in the ith protein. We decided to use the Kunitz domain (PF00014) as a model for the proteins and set all $N_i = 53$. The 53×53 submatrices that define the contacts within each protein were defined by extracting the contacts of the PDB structure 5pti of the Kunitz domain. This implies that the internal structure of every protein is the same.

We defined as contacting proteins the protein pairs $1-2, 2-3, 3-4, 4-5$ and $1-5$. For the 53×53 submatrices that define the contacts between contacting protein pairs we used random binary matrices with 10% of the number of internal contacts. This was done individually for each contacting protein pair such that no two contact matrices between two proteins were the same. For non-contacting protein pairs all entries of the contact matrices were set to 0.

The resulting contact map can be seen in Fig. E.

2) Couplings for every contact in the contact map were defined. As a basis for this, couplings and fields inferred from the PF00014 PFAM alignment (Kunitz Domain) were used. This inference was done using a masking with the PDB structure, such that only couplings corresponding to PDB-contacts were allowed to differ from zero. Given that the same PDB-contacts were used to define the contacts within one protein in the artificial complex, we could use the couplings thus inferred without change for the couplings within the artificial proteins.

Then we defined the couplings for residue contacts between two proteins. For every such a resiue contact we chose randomly a coupling of an internal contact as inferred from the Kunitz domain alignment and assigned it to the residue contact.

Notice that the 'coupling' between two sites i and j is actually a 21×21 matrix $J_{ij}(a, b)$ where a and b can be any of the 21 amino acids. Given that the internal structure of these matrices might be important we decided to treat the matrices J_{ij} as single entities and not change their internal structure.

The fields for every residue, a vector of length 21 for every of the $5 \cdot 53$ residues, were randomly chosen from the inferred fields.

From these couplings and fields, sequences were generated by MC (see section below) and inferred by plmDCA. Interestingly, a crude comparison between the histogram of the scores in the artificial model seem to be very close to that obtained for instance for the LRU case as shown in Fig. B.

In Table M we compare the ranks of the strongest inter-protein residue interaction scores in the generating model and the inferred model. The first column represents the rank of the inter-protein residue interaction in the generating model, the second column the rank of the same residue interaction in the inferred model. The model was inferred with the combined strategy and with 4000 sequences. The numbering is

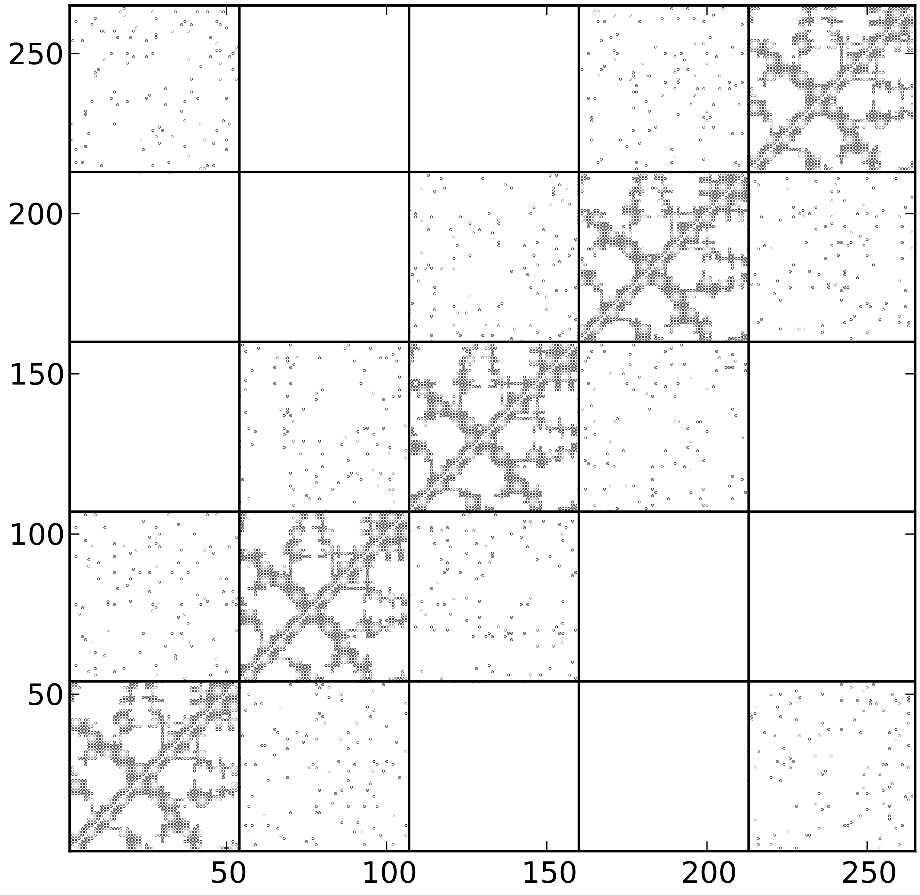

Figure E: Contact map of the artificial protein complex

Original Rank	Inferred Rank
1	101
2	13806
3	10658
4	64
5	4
6	9575
7	1
8	15890
9	6712
10	1035
7	1
32	2
41	3
5	4
11	5
11473	6
22464	7
53	8
1877	9
26	10

Table M: Original vs. inferred rank for the 10 largest original inter-protein residue interaction scores and the 10 largest inferred inter-protein residue interaction scores

treating the complex as one large protein.

E.1. Monte Carlo Sequence Generation

Given the parameters of the artificial model, a simple MCMC algorithm was run to generate samples from the corresponding distribution. We used one million MC steps to equilibrate the chain and took a sample every one million steps.

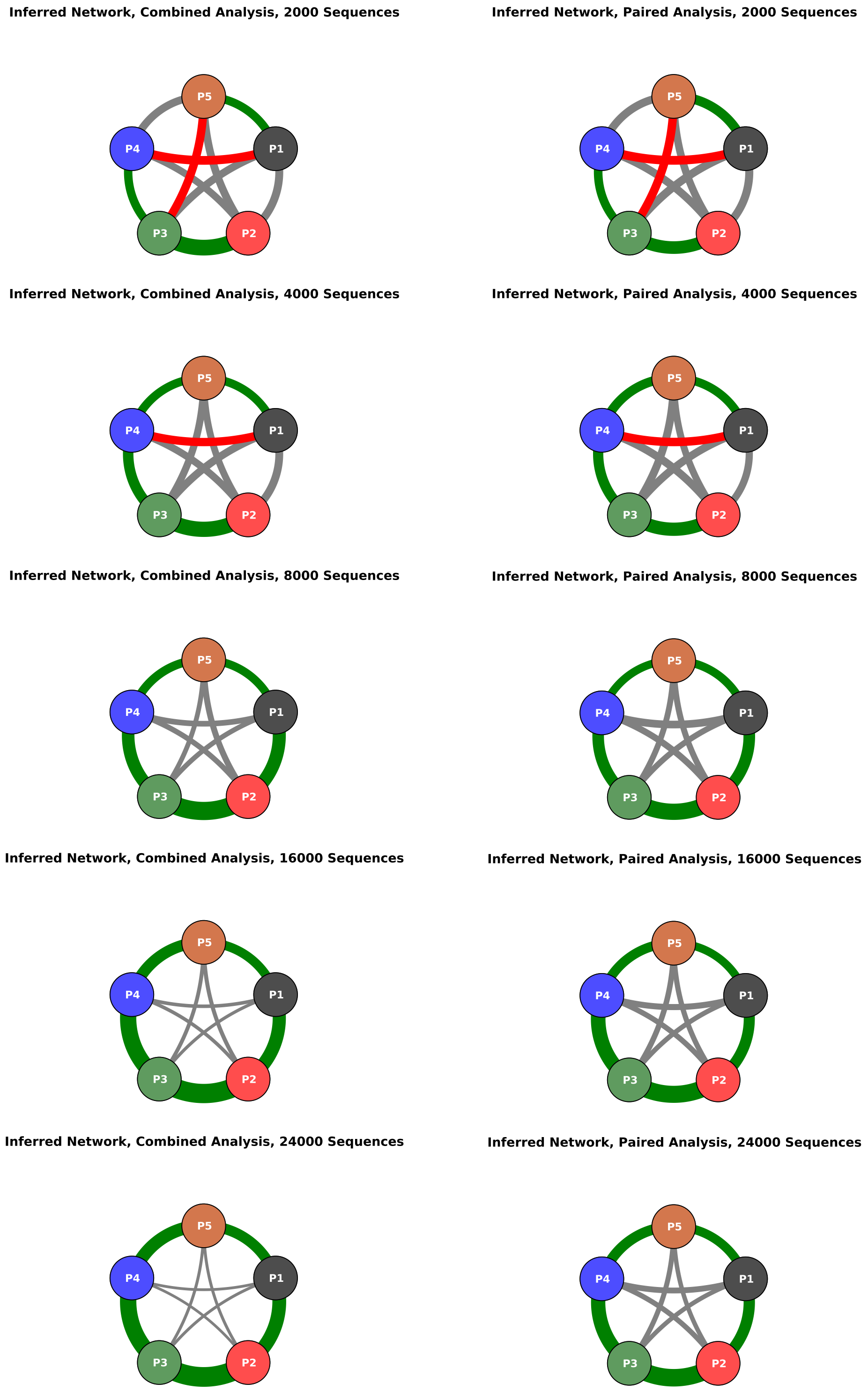

Figure F: Inferred protein network for different sample sizes; the line-thickness is proportional to the inferred interaction scores between the proteins (mean of the 4 highest residue interaction scores). The thickness has been normalized in the sense that the scores have been divided by the mean of the scores of the network. The color code is applied for the first 5 predictions and shows a green line if the prediction is a true positive and a red line if the prediction is a false positive. Predictions after the first 5 are grey.

Combined Analysis: The complete sequences in their whole length were used for the inference and calculation of the scores

Paired Anlysis: Every protein family was independently cut out of the generated sequences and thus a MSA for only this protein created. These single MSAs were then paired for all protein pairs and used for inference and calculation of the scores.

F. Large scale network inference

In order to test the approach on a larger scale we created all possible protein pairs from all proteins in the ribosome and the trp operon. The matching procedure was identical to the procedure used in the individual systems.

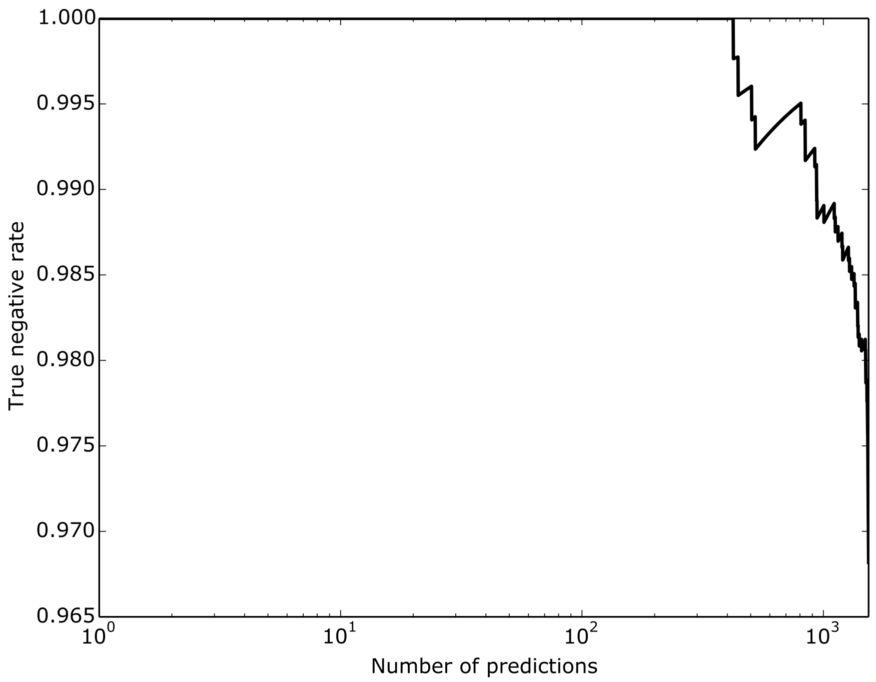

Figure G: True negative rate; all possible protein pairs between RS,RL and Trp proteins are considered and the protein-protein interaction score is defined as the average of the 4 largest interaction scores on the residue level (as in the main paper). The true negative rate is the fraction of true negatives in the N pairs with the lowest interaction score, where N is the value indicated by the x-axis.

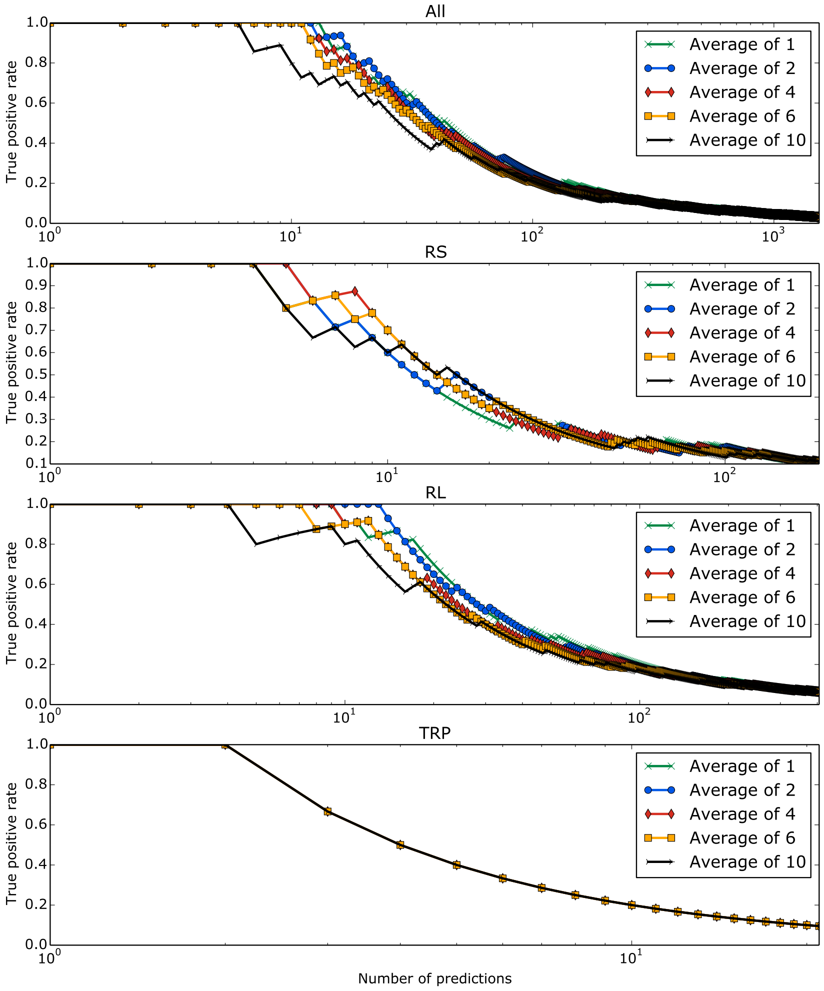

Figure H: True positive rates at a given number of predictions; All: All possible protein pairs between RS, RL and Trp proteins are considered; RS: Protein pairs within the small ribosomal subunit; RL: Protein pairs within the large ribosomal subunit; Trp: Protein pairs of the Trp operon. Different lines indicate a different number of averaged inter-protein scores on the residue level to get a protein-protein interaction score

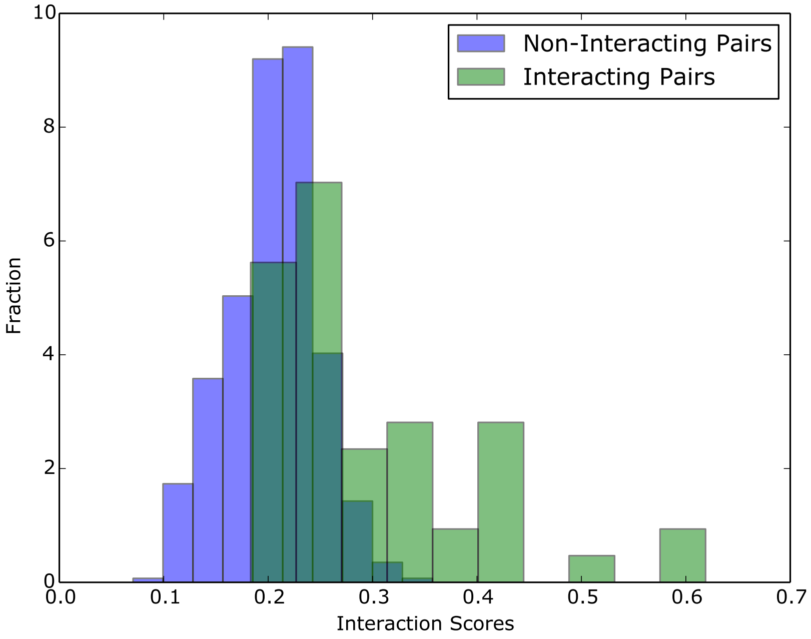

Figure I: Histograms of interaction scores in the network comprising all possible protein pairs between RS, RL and Trp proteins. The protein-protein interaction scores were calculated averaging the 4 largest inter-protein residue interaction scores (as in the main paper). The histogram shows true positives and true negatives seperately. Both histograms are normalized.

References

[1] S. Balakrishnan, H. Kamisetty, J. G. Carbonell, S. I. Lee, and C. J. Langmead. Learning generative models for protein fold families. *Proteins: Struct., Funct., Bioinf.*, 79:1061, 2011.

[2] Carlo Baldassi, Marco Zamparo, Christoph Feinauer, Andrea Procaccini, Riccardo Zecchina, Martin Weigt, and Andrea Pagnani. Fast and accurate multivariate gaussian modeling of protein families: Predicting residue contacts and protein-interaction partners. *PLoS ONE*, 9(3):e92721, 2014.

[3] Jeff Bezanzon, Stefan Karpinski, Viral Shah, and Alan Edelman. Julia: A fast dynamic language for technical computing. In *Lang.NEXT*, April 2012.

[4] Lukas Burger and Erik Van Nimwegen. Accurate prediction of protein−protein interactions from sequence alignments using a bayesian method. *Molecular Systems Biology*, 4(165):165, 2008.

[5] The UniProt Consortium. Uniprot: a hub for protein information. *Nucleic Acids Research*, 43(D1):D204–D212, 2015.

[6] M. Ekeberg, C. Lövkvist, Y. Lan, M. Weigt, and E. Aurell. Improved contact prediction in proteins: Using pseudolikelihoods to infer potts models. *Physical Review E*, 87(1):012707, 2013.

[7] Magnus Ekeberg, Tuomo Hartonen, and Erik Aurell. Fast pseudolikelihood maximization for direct-coupling analysis of protein structure from many homologous amino-acid sequences. *arXiv preprint arXiv:1401.4832*, 2014.

[8] Robert D. Finn, Alex Bateman, Jody Clements, Penelope Coggill, Ruth Y. Eberhardt, Sean R. Eddy, Andreas Heger, Kirstie Hetherington, Liisa Holm, Jaina Mistry, Erik L. L. Sonnhammer, John Tate, and Marco Punta. Pfam: the protein families database. *Nucleic Acids Research*, 42(D1):D222–D230, 2014.

[9] Robert D. Finn, Jody Clements, and Sean R. Eddy. Hmmer web server: interactive sequence similarity searching. *Nucleic Acids Research*, 39(suppl 2):W29–W37, 2011.

[10] D. T. Jones, D. W. A. Buchan, D. Cozzetto, and M. Pontil. PSICOV: precise structural contact prediction using sparse inverse covariance estimation on large multiple sequence alignments. *Bioinformatics*, 28:184, 2012.

[11] Kazutaka Katoh and Daron M. Standley. Mafft multiple sequence alignment software version 7: Improvements in performance and usability. *Molecular Biology and Evolution*, 30(4):772–780, 2013.

[12] Debora S. Marks, Lucy J. Colwell, Robert Sheridan, Thomas A. Hopf, Andrea Pagnani, Riccardo Zecchina, and Chris Sander. Protein 3d structure computed from evolutionary sequence variation. *PLoS ONE*, 6(12):e28766, 12 2011.

[13] MATLAB. *version R2014a.* The MathWorks Inc., Natick, Massachusetts, 2014.

[14] Faruck Morcos, Andrea Pagnani, Bryan Lunt, Arianna Bertolino, Debora S Marks, Chris Sander, Riccardo Zecchina, José N Onuchic, Terence Hwa, and Martin Weigt. Direct-coupling analysis of residue coevolution captures native contacts across many protein families. *Poc. Natl. Acad. Sci.*, 108(49):E1293–E1301, 2011.

[15] Martin Weigt, Robert A White, Hendrik Szurmant, James A Hoch, and Terence Hwa. Identification of direct residue contacts in protein–protein interaction by message passing. *Poc. Natl. Acad. Sci.*, 106(1):67–72, 2009.

[16] Martin Weigt, Robert A. White, Hendrik Szurmant, James A. Hoch, and Terence Hwa. Identification of direct residue contacts in protein-protein interaction by message passing. *Poc. Natl. Acad. Sci.*, 106(1):67–72, 2009.
